# Prevalence and significance of potential drug-drug interactions among cancer patients receiving chemotherapy

**DOI:** 10.1186/s12885-020-06855-9

**Published:** 2020-04-19

**Authors:** Mohammad Ismail, Sehrash Khan, Fahadullah Khan, Sidra Noor, Hira Sajid, Shazia Yar, Irum Rasheed

**Affiliations:** Department of Pharmacy, University of Peshawar, Peshawar, Khyber Pakhtunkhwa Pakistan

**Keywords:** Patient safety, Cancer, Supportive therapy, Potential drug-drug interactions, Polypharmacy

## Abstract

**Background:**

Cancer patients often receive multiple drugs to maximize their therapeutic benefit, treat co-morbidities and counter the adverse effects of chemotherapy. Concomitant administration of multiple drugs increases the risk of drug interactions leading to compromised therapeutic efficacy or safety of therapy. The purpose of this study was to identify the prevalence, levels and predictors of potential drug-drug interactions (pDDIs) among cancer patients.

**Methods:**

Six hundred and 78 patients receiving chemotherapy from two tertiary care hospitals were included in this cross-sectional study. Patient medication profiles were screened for pDDIs using the Micromedex® database. Logistic regression analysis was performed to identify the predictors of pDDIs.

**Results:**

The overall prevalence of pDDIs was 78%, majority of patients had 1–2 pDDIs (39.2%). A total of 1843 pDDIs were detected. Major-pDDIs were most frequent (67.3%) whereas, a significant association of pDDIs was found between > 7 all prescribed drugs (*p* < 0.001) and ≥ 3 anti-cancer drugs (*p* < 0.001). Potential adverse outcomes of these interactions include reduced therapeutic effectiveness, QT interval prolongation, tendon rupture, bone marrow suppression and neurotoxicity.

**Conclusions:**

Major finding of this study is the high prevalence of pDDIs signifying the need of strict patient monitoring for pDDIs among cancer patients. Patients at higher risk to pDDIs include those prescribed with > 7 any types of drugs or ≥ 3 anticancer drugs. Moreover, list of most frequently identified major and moderate interactions will aid health care professional in timely identification and prevention of pDDIs.

## Background

The global cancer burden is on the rise due to increased prevalence of risk factors such as smoking, environmental pollution, obesity, unhealthy diet, physical inactivity, infections (hepatitis, helicobacter pylori and human papilloma virus) and use of oral contraceptives [1–4]. The Global burden of cancer (GLOBOCAN) 2012, reported an estimated 14.1 million new cases of cancer, which is anticipated to rise by 70% over the next 20 years [2, 3]. Cancer is equally prevalent around the world [3, 5], however differences in the pattern of cancer and its subsequent therapy exist from region to region [5]. The overall survival of cancer is high in developed countries due to timely and easy access to standard health care facilities [1, 2, 6, 7].

The use of cytotoxic agents is inevitable for the cure of cancer despite the availability of a number of other alternatives such as surgery and radiation [8, 9]. The main aim of chemotherapy is to cure cancer, extend life years and improve the overall quality of life [7, 8]. Cytotoxic agents are often administered in combination containing two or more cytotoxic drugs as multiple drug regimens along with other medicines to achieve maximum therapeutic benefit and counter the adverse effects of chemotherapy or to treat other co-illnesses [10, 11]. However, interpatient variability is frequently observed especially with oral cytotoxic agents due to drug interactions as a result of polypharmacy [12, 13].

Drug-drug interactions (DDIs) are drug combinations that may result in therapeutic failure or potentially serious adverse events than from solitary administration [14–18]. An estimated 2% of hospital admissions are due to adverse effects caused by DDIs [19, 20] and approximately 4% of the cancer patients die because of adverse effects caused by drug interactions [21] however, DDIs are often predictable and preventable [22, 23]. Advance age, prolonged hospital stay and increased number of prescribed medicines are strong predictors of DDIs [24–27]. The risk of DDIs and its associated adverse events are higher in cancer patients as they frequently receive multiple drugs concomitantly [17, 25]. Moreover, there has been a recent increase in the availability and use of anticancer agents because of favorable therapeutic outcome and cost effectiveness [12, 28].

DDIs among cancer patients have been well studied in developed countries [20, 24, 26–29]. However, such studies have been poorly addressed in developing countries like Pakistan. There are a few studies which have their own scope and limitations such as involving only QT prolonging DDIs [30], small sample size and different drug interactions screening tool [31], and primarily focused on medication safety [32]. Further, we cannot generalize the findings of developed countries to the developing countries because of variations in drug prescribing pattern, utilization of anticancer drugs, pattern of cancers, drug interactions screening before prescribing, and non-availability of standard healthcare services.

DDIs involving anticancer drugs are a major concern in oncology practice due to their potential to cause severe adverse effects [1, 28, 33, 34]. Moreover, knowledge about the most common interacting drugs used in cancer patients and identification of predictors of pDDIs are essential to reduce avoidable drug-related problems and increase the efficacy and compliance of chemotherapy [14, 17]. Additionally, this study will be helpful for the promotion of rational drug use and for the prevention and management of drug interactions leading to improved therapeutic outcome and patients’ quality of life [34, 35]. Therefore, the study aimed to identify the prevalence, levels, predictors and potential adverse outcome of pDDIs among cancer patients receiving chemotherapy.

## Methods

### Study settings and design

A cross-sectional study was performed in two tertiary care hospitals of Peshawar, Khyber Pakhtunkhwa, Pakistan: Hayatabad Medical Complex (HMC) and Northwest General Hospital and Research Center (NWGH & RC). HMC is a public sector tertiary care teaching hospital whereas, NWGH & RC is a private sector hospital.

### Selection criteria

Patients of any age and either gender diagnosed with any type of cancer and treated with anticancer agents (either intravenous and/ or oral) were included in this study. Whereas, patient profiles were excluded if the required information were lacking.

### Data collection and screening for pDDIs

Data regarding patient’s demographic, symptoms, laboratory results and prescribed medications were collected after acquiring written permission from the administration of respective hospitals.

Micromedex Drug-Reax® (Truven Health Analytics, Greenwood Village, Colorado, USA) was used for the screening of patients’ medication profile for pDDIs [36]. We select this software because it has got highest sensitivity and specificity score [37, 38]. Further, it has got sensitivity score of 70% in identifying drug interactions involving oral anticancer drugs [39]. According to the description of this database, all detected interactions were categorized on the basis of severity-levels and documentation-levels [36]. All available lab values were reviewed to identify abnormal results.

### Statistical analysis

In statistical analysis, quantitative data were presented as frequencies and percentages. Logistic regression analysis was applied in order to identify association of pDDIs presence with patients’ gender, age, prescribed medications, hospitalization status, cancer type, presence of metastasis, treatment type and treatment intent. For each predictor odds ratio (OR) a 95% confidence intervals (CIs) was determined by performing univariate logistic regression analysis. For variables with significant univariate *p*-values multivariate analysis was performed. In this study *p*-value of 0.05 or less was considered significant. SPSS version-23 was used for statistical analysis.

## Results

### Patients characteristics

Out of total 678 patients, 358 (52.8%) were male and 320 (47.2%) were female. Majority of patients were in the age range of 41–60 years (29.5%) followed by 21–40 years (26.4%). The use of 7–9 drugs (37.3%) and 10–12 drugs (29.4%) were most frequent. Whereas, majority of patients (81.9%) were prescribed ≥2 anti-cancer drugs and ≥ 4 supportive drugs (87.6%) as presented in Table 1.

**Table 1:** Patient characteristics (*N* = 678)

Variables	Patients: n (%)
**Gender**	
Male	358 (52.8)
Female	320 (47.2)
**Age (years)**	
≤ 10	101 (14.9)
11–20	110 (16.2)
21–40	179 (26.4)
41–60	200 (29.5)
> 60	88 (13)
**All prescribed drugs**	
≤ 6	120 (17.7)
7–9	253 (37.3)
10–12	199 (29.4)
13–15	64 (9.4)
> 15	42 (6.2)
**Anticancer drugs**	
1	123 (18.1)
2	264 (38.9)
3	159 (23.5)
≥ 4	132 (19.5)
**Supportive care drugs**	
≤ 3	84 (12.4)
4–6	242 (35.7)
7–9	246 (36.3)
≥ 10	106 (15.6)

### Cancer profile and treatment of study subjects

Table 2 illustrates cancer profile and its treatment for study subjects. Solid malignancies were most frequent among the study participants (53.1%) as compared with hematologic malignancies (46.9%). Metastasis was seen in 112 (16.5%) patients whereas 620 (91.4%) patients were receiving curative treatment. Moreover, the use of cytotoxic agents (74.9%) and combination therapy (24%) were common while hormonal or monoclonal agents were rarely prescribed. The most frequent solid malignancies include gastrointestinal cancer (13.1%), breast cancer (9.4%), gynecological cancer (6.9%), musculoskeletal cancer (6.2%) and genitourinary cancer (6%). Likewise, the most frequent hematological malignancies include acute lymphoblastic leukemia (18.7%), non-Hodgkin lymphoma (17.3%), and acute myelogenous leukemia (4.1%).

**Table 2:** Cancer characteristics and their types

Variables	Patients: n (%)
**Cancer type**	
Solid malignancy	360 (53.1)
Hematologic cancer	318 (46.9)
**Metastasis**	
Present	112 (16.5)
Absent	566 (83.5)
**Treatment intent**	
Curative	620 (91.4)
Palliative	58 (8.6)
**Type of chemotherapy**	
Cytotoxic agents	508 (74.9)
Hormonal agents	4 (0.6)
Monoclonal agents	3 (0.4)
Combination^a^	163 (24)
**Solid malignancy**	
Gastrointestinal cancer	89 (13.1)
Breast cancer	64 (9.4)
Gynecological cancer	47 (6.9)
Musculoskeletal cancer	42 (6.2)
Genitourinary cancer	41 (6)
Head and neck cancer	19 (2.8)
Neurological cancer	13 (1.9)
Respiratory cancer	11 (1.6)
Others	34 (5)
**Hematological malignancy**	
Acute lymphoblastic leukemia	127 (18.7)
Non-Hodgkin lymphoma	117 (17.3)
Acute myelogenous leukemia	28 (4.1)
Chronic lymphocytic leukemia	17 (2.5)
Hodgkin lymphoma	18 (2.5)
Chronic myelogenous leukemia	7 (1)
Others	5 (0.7)

-^a^Combination means regimen comprising of cytotoxic, hormonal or monoclonal agents in combination

### Prevalence of pDDIs

**Fig. 1:**
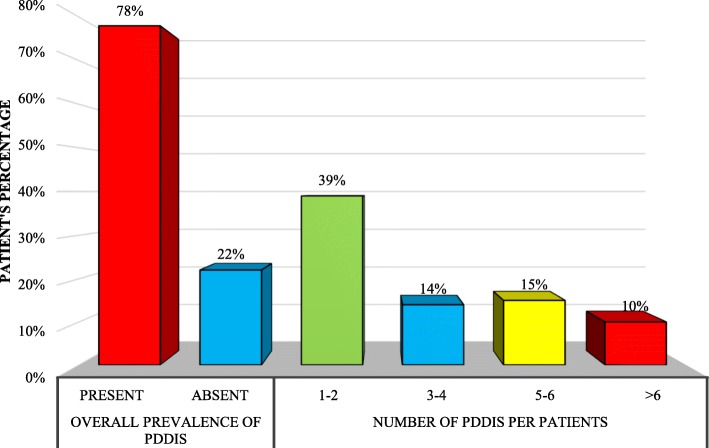
Prevalence of pDDIs. -PDDIs = potential drug-drug interactions. -Overall prevalence means occurrence of pDDIs regardless of severity. -Percentages do not add up to 78% because many patients were exposed to multiple pDDIs of different severities

Figure 1 indicates that 529 patients were exposed to at least one pDDI (overall prevalence = 78%). Majority of patients had 1–2 pDDIs (266), 5–6 pDDIs (100) and 3–4 pDDIs (93).

### Levels of pDDIs

**Fig. 2:**
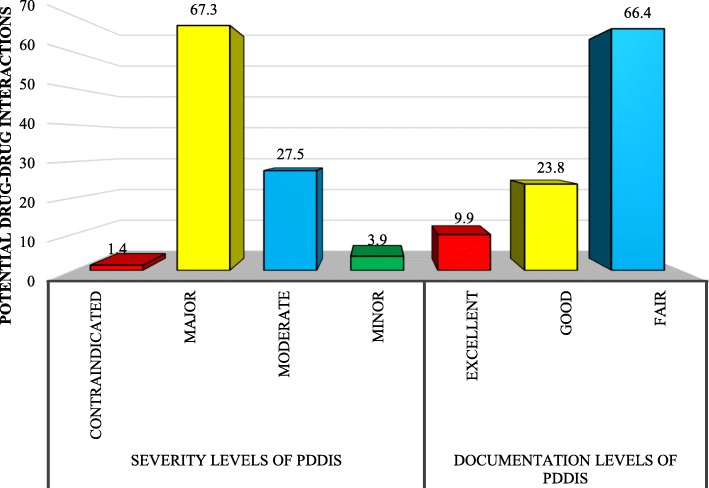
Levels of pDDIs

Overall, 1843 pDDIs were detected, of which, 1240 (67.3%) were of major and 507 (27.5%) were of moderate severity while contraindicated pDDIs were least frequent accounting for 25 (1.4%) pDDIs. The documentary evidence of majority of pDDIs were fair (66.4%) and good (23.8%) (Fig. 2).

### Predictors of pDDIs

Table 3 demonstrates the results of univariate and multivariate logistic regression analysis for exposure to pDDIs. Results of univariate logistic regression analysis indicates a significant relation of pDDIs with all prescribed drugs, anticancer drugs, supportive care drugs, hospitalization, type of cancer and type of cancer treatment. The odds of pDDIs are 3.6 times with > 7 all prescribed drugs (*p* < 0.001), 4.7 times with ≥3 anti-cancer drugs (*p* < 0.001), 1.9 times with > 3 supportive care drugs (*p* = 0.001), 1.8 times with hospitalization (*p* = 0.004), 2.1 times with combination treatment (*p* = 0.003) and 0.4 times with solid malignancy (*p* < 0.001). Whereas, the risk is insignificant for gender, age, presence of metastasis and treatment intent.

**Table 3:** Univariate and multivariate logistic regression analysis

Variables	Univariate		Multivariate	
	OR (95% CI)	*p*-value	OR (95% CI)	*p*-value
**Gender**				
Male	Reference		–	
Female	0.7 (0.5–1)	0.108	–	–
**Age (years)**				
≤ 50	Reference		–	
> 50	0.8 (0.5–1.2)	0.318	–	–
**All drugs prescribed**				
≤ 7	Reference		Reference	
> 7	3.6 (2.5–5.3)	0.0001	3.5 (2.2–5.5)	0.0001
**Anticancer drugs**				
≤ 2	Reference		Reference	
≥ 3	4.7 (3–7.4)	0.0001	3.6 (2.1–6.2)	0.0001
**Supportive care drugs**				
≤ 3	Reference		Reference	
> 3	1.9 (1.3–2.9)	0.001	0.6 (0.3–1.2)	0.161
**Hospitalization status**				
Ambulatory	Reference		Reference	
Hospitalized	1.8 (1.2–2.6)	0.004	1.3 (0.8–2)	0.327
**Cancer type**				
Hematological malignancy	Reference		Reference	
Solid malignancy	0.4 (0.3–0.6)	0.0001	0.7 (0.4–1.1)	0.173
**Metastasis**				
Present	Reference		–	
Absent	1.5 (0.9–2.4)	0.07	–	–
**Treatment type**				
Cytotoxic agents	Reference		Reference	
Combination drugs^a^	2.1 (1.3–3.4)	0.003	0.7 (0.4–1.3)	0.343
**Treatment intent**				
Curative	Reference		–	
Palliative	1.6 (0.8–3.3)	0.218	–	–

- *pDDIs* Potential drug–drug interactions; *OR* Odds ratio; *CI* Confidence interval

-Hosmer–Lemeshow goodness-of-fit test: *p* = 0.3

**-**^a^Combination means regimen comprising of cytotoxic, hormonal or monoclonal agents in combination

Likewise, the results of multivariate logistic regression analysis show a significant relation of pDDIs with the presence of > 7 all prescribed drugs (*p* < 0.001) and ≥ 3 anti-cancer drugs (*p* < 0.001). Whereas, the association of pDDIs with supportive care drugs (*p* = 0.2), hospitalization (*p* = 0.3), type of cancer (*p* = 0.2) and type of treatment (*p* = 0.3) are insignificant.

### Abnormal symptoms and laboratory results

Table 4 presents the abnormal biochemical results and symptoms among study subjects. Hematological tests show reduced hemoglobin in majority of patients (58.7%) whereas, 118 (17.4%) patients were reported with reduced red blood cell count. Similarly, leukocytopenia was observed in 96 (14.2%) patients with decreased neutrophil count in 70 (10.3%) patients, decreased lymphocytes in 149 (21.9%) patients and reduced eosinophils and monocytes in 47 (6.9%) and 372 (54.9%) patients, respectively. Abnormal high serum creatinine was reported in 26 (3.8%) patients and 12 (1.8%) patients had elevated bilirubin level. While among the liver function tests, 121 (17.8%) patients had elevated ALT levels and 106 (15.6%) patients had elevated alkaline phosphatase level. Whereas, the most frequently observed symptoms among the study participants include fever (12.1%), generalized body ache (9.3%), nausea & vomiting (4.7%), abdominal pain (4.1%) and cough (3.8%).

**Table 4:** Abnormal biochemical results and symptoms among study subjects

Laboratory test	Patients: n (%)
**Hematological tests**	
Hemoglobin	
< 12 g/dL	398 (58.7)
Red blood cells	
< 4 × 106/mm3	118 (17.4)
> 5.5 × 106/mm3	11 (1.6)
Total leukocyte count	
< 4000 /mm3	96 (14.2)
> 11,000 /mm3	145 (21.4)
Platelet count	
< 150,000 /mm3	138 (20.4)
> 450,000 /mm3	63 (9.3)
Neutrophils	
< 40%	70 (10.3)
> 75%	113 (16.7)
Lymphocytes	
< 20%	149 (21.9)
> 45%	91 (13.4)
Eosinophils	
< 1%	47 (6.9)
> 6%	17 (2.5)
Monocytes	
< 6%	372 (54.9)
> 10%	23 (3.4)
**Renal function tests**	
Serum creatinine	
> 1.3 mg/dl	26 (3.8)
Total bilirubin > 1.5 mg/dl	12 (1.8)
**Liver function tests**	
Alanine aminotransferase	
> 40 U/L	121 (17.8)
Aspartate aminotransferase	
> 40 U/L	46 (6.8)
Alkaline phosphatase	
< 35 U/L	3 (0.4)
> 130 U/L	106 (15.6)
**Symptoms**	
Fever	82 (12.1)
Generalized body aches	63 (9.3)
Nausea and vomiting	32 (4.7)
Abdominal pain	28 (4.1)
Cough	26 (3.8)
Anorexia	19 (2.8)
Shortness of breath	15 (2.2)
Generalized weakness	11 (1.6)
Pallor	11(1.6)
Swelling in different body parts	10 (1.5)
Bleeding from different body parts	9 (1.3)
Loose motions	10 (1.3)
Abdominal distension	8 (1.2)
Epigastric pain	9 (1.2)
Dysphagia	10 (1.2)
Sweating	11 (1.2)
Urinary tract infections	12 (1.2)

### Wide spread interacting drug combinations

Most frequently detected pDDIs are enlisted in Table 5 along with their severity, documentation levels and potential adverse outcomes. Reduced therapeutic effectiveness, QT interval prolongation, drug toxicity such as tendon rupture, bone marrow suppression, seizures, serotonin syndrome, neurotoxicity and cardiomyopathy were the potential adverse outcomes of these interactions. Potential drug-drug interactions involving anti-cancer agents are enlisted in additional Table 1.

**Table 5:** Most frequent pDDIs among cancer patients

Drug-drug interaction	Frequency	Severity	Evidence	Potential adverse outcome
Dexamethasone + Vincristine	228	Major	Fair	Decreased vincristine plasma concentration.
Doxorubicin + Dexamethasone	164	Major	Fair	Reduced doxorubicin exposure.
Ondansetron + Prochlorperazine	116	Major	Fair	Increased risk of QT interval prolongation.
Cyclophosphamide + Doxorubicin	105	Major	Fair	High risk of cardiomyopathy.
Ciprofloxacin + Dexamethasone	102	Moderate	Excellent	Increased risk for tendon rupture.
Ciprofloxacin + Ondansetron	89	Major	Fair	Increased risk of QT interval prolongation.
Ciprofloxacin + Prochlorperazine	83	Major	Fair	Increased risk of QT interval prolongation.
Cyclophosphamide + Ondansetron	75	Moderate	Good	Decreased cyclophosphamide systemic exposure.
Allopurinol + Cyclophosphamide	66	Major	Good	Cyclophosphamide toxicity (bone marrow suppression, nausea, vomiting).
Metoclopramide + Tramadol	48	Major	Fair	Increased risk of seizures.
Ciprofloxacin + Doxorubicin	33	Major	Fair	Increased doxorubicin exposure.
Calcium Chloride + Ciprofloxacin	32	Moderate	Good	Decreased ciprofloxacin efficacy.
Ondansetron + Tramadol	31	Moderate	Excellent	Reduced efficacy of tramadol.
Tropisetron + Tramadol	24	Major	Fair	Increased risk of serotonin syndrome.
Fluorouracil + Leucovorin	23	Moderate	Good	Increased concentrations of 5-fluorouracil and fluorouracil toxicity (granulocytopenia, anemia, thrombocytopenia, stomatitis, vomiting).
Asparaginase + Vincristine	19	Major	Fair	Increased vincristine exposure causing neurotoxicity.
Cisplatin + Docetaxel	14	Moderate	Excellent	Increased risk of neuropathy.
Methotrexate + Omeprazole	13	Major	Good	Increased concentration of methotrexate and its metabolite and an increased risk of methotrexate toxicity.
Cisplatin + Doxorubicin	11	Major	Good	Increased risk of Secondary malignancy i.e. secondary leukemia.
Fluconazole + Metronidazole	10	Major	Fair	Increased risk of QT interval prolongation and arrhythmias.

## Discussion

This study presents the frequency, severity and predictors for pDDIs and list of most frequent pDDIs among cancer patients undergoing chemotherapy. An overall 78% prevalence of pDDIs is higher in comparison with other studies conducted in oncology setting. A study from Iran reported 62.8% prevalence of pDDIs in patients with hematological malignancy [40]. Another study from Netherland reported a prevalence rate of 46% among patients using oral anticancer drugs [41]. Whereas, a study from the United States of America reported 40% prevalence rate of pDDIs [29]. Similarly, the prevalence rate of present study is higher in comparison with studies from other specialties such as internal medicine (52.8%) [25], psychiatry (64.8%) [42], pediatrics (25.8%) [43] and pulmonology (45%) [44]. Such widespread variability in prevalence may be attributed to differences in study designs, inclusion and exclusion criteria, study population & their characteristics, study settings, presence or absence of clinical pharmacy services, prescribing pattern, drugs involving, and high sensitivity of drug interactions screening databases/sources. The high prevalence of pDDIs identified in our study demands thoughtfulness regarding the issue of pDDIs in cancer patients receiving chemotherapy.

Levels of pDDIs are imperative for healthcare professionals to evaluate their potential clinical significance and rationalize the patients’ treatment. All interactions are not equally harmful, therefore, classification of the identified interactions into different levels helps in proper management of these interactions. The more frequent occurrence of major pDDIs is an important finding of this study, necessitating the need of strict monitoring of patients as these interactions carry higher potential for causing life threatening adverse reactions. A study reported similar results in hematological malignancies [40] however, in majority of studies moderate pDDIs are more frequent [41, 45, 46].

The use of polypharmacy is prevalent among cancer patients. The significant association of > 7 prescribed drugs and ≥ 3 anti-cancer drugs with pDDIs in present study are coherent with other studies both in oncology setting and other specialties [17, 22, 27, 47]. The presence of polypharmacy in cancer patients demands the screening of prescribed medications for timely prediction and prevention or minimization of any unwanted negative consequences as polypharmacy is inevitable among cancer patients. The univariate analysis estimated a significant association of pDDIs with hospitalized patients, combination chemotherapy and patients having solid malignancy however, they were insignificant in multivariate analysis. Moreover, like other studies age, gender, metastasis and treatment intent had insignificant association with pDDIs [17, 34, 45] however, few studies have reported a significant association of pDDIs with gender, age and type of cancer [28, 34, 48, 49].

List of most frequent pDDIs particularly those of contraindicated, major and moderate severity are of utmost importance for health care providers. It can aid in the selective screening of pDDIs by overburdened health care professionals. Such information is needed for health care professionals to estimate the risk in specific patients and guide their therapeutic decision making [8]. Patients at risk of these interactions may be given special attention and their therapy may be closely monitored for any potential adverse effect.

There are a few potential limitations of this study. Although, this work explored pharmacoepidemiology of pDDIs in cancer patients, the exact extent of patient suffering due to these interactions were not studied. The study was conducted only in two hospitals, which is the second point which may limit the generalizability of this study. Moreover, only one drug interactions screening database (Micromedex Drug-Reax®) was used for the identification of pDDIs, however, other sources are also available which may not necessarily give the same results. Further, we have only included hospitalized cancer patients receiving intravenous and/ or oral anticancer agents. In hospital settings, mostly intravenous anticancer therapy is provided. We don’t include cancer patients treated in homebased care settings which could provide a different DDIs pattern due to frequent use of oral anticancer agents.

## Conclusions

This study points out a high prevalence of pDDIs among cancer patients treated with anti-cancer agents. Majority of interactions were of major and moderate severity. Patients with polypharmacy i.e. > 7 all prescribed drugs or ≥ 3 anticancer drugs had a significantly increased risk of pDDIs. Whereas, list of most frequently identified major and moderate interactions will aid in timely identification, prevention and management of pDDIs and their adverse outcome in cancer patients. Moreover, strict patient monitoring is recommended especially in patients with > 7 all prescribed drugs or ≥ 3 anticancer drugs for timely prevention and/or management of negative clinical outcomes associated with these interactions particularly those involving cytotoxic drugs.

## Data Availability

The datasets used and/or analyzed during the current study are available from the corresponding author on reasonable request.

## Supplementa0ry information

**Additional file 1: Table S1.** PDDIs of major severity involving anticancer drugs.

## Abbreviations

CI: Confidence interval
DDIs: Drug-drug interactions
HMC: Hayatabad medical complex
NWGH & RC: Northwest general hospital and research center
OR: Odds ratios
pDDIs: Potential drug-drug interactions
