# Supplementary material for: Prevalence and significance of potential drug-drug interactions among cancer patients receiving chemotherapy
Source: BMC Cancer. 2020 Apr 19;20:335. doi: 10.1186/s12885-020-06855-9 (PMC7168989; doi:10.1186/s12885-020-06855-9)
Supplement: Supplementary file 1 — Additional file 1: Table S1. PDDIs of major severity involving anticancer drugs. [file 12885_2020_6855_MOESM1_ESM.docx]

**Additional Table 1** PDDIs of major severity involving anticancer drugs

| **Major pDDIs** | **Frequency (n)** | **Percentage (%)** |
| --- | --- | --- |
| Dexamethasone + Vincristine | 228 | 33.6 |
| Doxorubicin + Dexamethasone | 164 | 24.2 |
| Cyclophosphamide + Doxorubicin | 105 | 15.5 |
| Allopurinol + Cyclophosphamide | 66 | 9.7 |
| Ciprofloxacin + Doxorubicin | 33 | 4.9 |
| Asparaginase + Vincristine | 19 | 2.8 |
| Methotrexate + Omeprazole | 13 | 1.9 |
| Cisplatin + Doxorubicin | 11 | 1.6 |
| Filgrastim + Vincristine | 10 | 1.5 |
| Fluconazole + Vincristine | 7 | 1 |
| Doxorubicin + Paclitaxel | 7 | 1 |
| Clarithromycin + Vincristine | 7 | 1 |
| Cisplatin + Furosemide | 7 | 1 |
| Esomeprazole + Methotrexate | 6 | 0.9 |
| Cisplatin + Paclitaxel | 5 | 0.7 |
| Metronidazole + Nilotinib | 2 | 0.3 |
| Methotrexate + Trimethoprim | 2 | 0.3 |
| Methotrexate + Sulfamethoxazole | 2 | 0.3 |
| Ibuprofen + Methotrexate | 2 | 0.3 |
| Doxorubicin + Warfarin | 2 | 0.3 |
| Cyclophosphamide + Trastuzumab | 2 | 0.3 |
| Vincristine + Warfarin | 1 | 0.1 |
| Nilotinib + Tramadol | 1 | 0.1 |
| Nilotinib + Tizanidine | 1 | 0.1 |
| Fluorouracil + Metronidazole | 1 | 0.1 |
| Fluorouracil + Methotrexate | 1 | 0.1 |
| Esomeprazole + Methotrexate | 1 | 0.1 |
| Erlotinib + Esomeprazole | 1 | 0.1 |
| Doxorubicin + Fluconazole | 1 | 0.1 |
| Cyclophosphamide + Warfarin | 1 | 0.1 |
| Ciprofloxacin + Erlotinib | 1 | 0.1 |
| Cimetidine + Fluorouracil | 1 | 0.1 |
| Capecitabine + Warfarin | 1 | 0.1 |
| Amoxicillin/Clavulanate + Methotrexate | 1 | 0.1 |
| Amitriptyline + Doxorubicin | 1 | 0.1 |
| pDDIs = Potential Drug-Drug Interactions | |  |
